# Chemical Forms and Health Risk of Cadmium in Water Spinach Grown in Contaminated Soil with An Increased Level of Phosphorus

**DOI:** 10.3390/ijerph16183322

**Published:** 2019-09-09

**Authors:** Chun-Ming Lam, Kuei-San Chen, Hung-Yu Lai

**Affiliations:** 1 Department of Post-Modern Agriculture, MingDao University, Changhua 52345, Taiwan; kenneth1227036@gmail.com; 2 Department of Soil and Environmental Sciences, National Chung Hsing University, Taichung 40227, Taiwan; s104039023@smail.nchu.edu.tw; 3 Innovation and Development Center of Sustainable Agriculture, National Chung Hsing University, Taichung 40227, Taiwan

**Keywords:** cadmium, chemical form, crop safety, phosphorus, risk assessment

## Abstract

(1) Background: Even in croplands with a low concentration of cadmium (Cd), there is still a risk for planting crops because of the high accumulation capacity of some leafy vegetables. (2) Methods: In this study, water spinach was planted in four main soil series (Wa, Eh, Tk, and Yu) in central Taiwan, which were spiked with Cd. The soil available phosphorous content was increased to 10–17 mg/kg, and the accumulation and developed chemical forms of Cd were analyzed. (3) Results: The experimental results showed that addition of phosphorous to Wa and Eh promoted the growth of water spinach. Accumulation and upward translocation of Cd were also increased in the phosphorus treatment compared with the control. The addition of phosphorus increased the percentage of Cd compartmentalized in undissolved Cd phosphate, which revealed that the mobility and toxicity of Cd were reduced in the phosphorus treatment. However, most of the water spinach was not edible because the vegetable-induced hazard quotient, which was calculated using three methods, showed hazardous potential in general.

## 1. Introduction

Cadmium (Cd) contamination of soil is a global problem associated with activities such as mining, leather treating, electroplating, and fertilization [1]. Plants have many mechanisms to alleviate stress due to Cd. Different plant organs use its chemical forms—including inorganic Cd (F_E_), water-soluble Cd (F_W_), pectate- and protein-integrated Cd (F_NaCl_), undissolved Cd phosphate (F_HAc_), Cd oxalate (F_HCl_), and residual Cd (F_R_)—in detoxification mechanisms [2,3]. In particular, the mobility of F_E_ and F_W_ is higher than that of other chemical forms; thus, it is easy to translocate them upward from roots to shoots [4,5,6]. The compartmentalization of Cd into chemical forms depends on the plant species under consideration [7,8], but many plants compartmentalize it into F_NaCl_ as a detoxification mechanism [5,7,9].

The application of phosphorous (P) enables changing the availability of heavy metals in soils [10] and thus decreases Cd accumulation in, for example, paddy rice [11]. However, the decrease in soil pH resulting from the application of P fertilizers increases the availability of Cd [12,13]. The application of P also results in the formation of Cd-P compounds, such as Cd_3_(PO_4_)_2_ [7,14], which could mitigate the toxicity of Cd [5], decrease the accumulation of Cd in plants [15,16], and promote plant growth [11]. However, Gao et al. [13] revealed that P treatment increased Cd accumulation in the roots and shoots of wheat as well as the P concentration in soil.

In this study, water spinach seeds were sown in samples of the four main soil series in central Taiwan, which were spiked with Cd solutions. The objective of this study was to understand the effect of increasing the soil available P concentration on the translocation of Cd from soil to different organs of plants based on changes in its chemical form. Besides the total concentration of Cd in the edible tissues of water spinach, the chemical form and artificial digesting agents extractable concentration of Cd were used to calculate the vegetable-induced hazard quotient (HQ_v_), which reflects the bioaccessible Cd fraction compared to the total concentration.

## 2. Materials and Methods

Four representative soil series in central Taiwan were collected, and their cation exchange capacity (CEC) [17], organic carbon content (OC) [18], and texture [19] were analyzed (Table 1). The soils used had different levels of available P content, including Wanho (Wa), Erhlin (Eh), Taikang (Tk), and Yuanlin (Yu). Ground soil samples were passed through 100-mesh stainless sieves, digested with aqua regia, filtered using filter papers (Whatman No. 42), and then the Cd concentration in the digestant was determined using a frame atomic absorption spectrophotometer (FAAS, Perkin Elmer AAnalyst 200). According to the Soil and Groundwater Pollution Remediation (SGWPR) Act of Taiwan, when the total Cd concentration in cropland soil is less than 2.5 mg/kg, it can be regarded as non-contaminated. To prepare the Cd-spiked soils (i.e., by increasing the total Cd concentration of these four soil series to near 2.5 mg/kg), 18.0 kg per soil series of ground soil samples were artificially spiked with solutions of cadmium nitrate tetrahydrate. Over three months prior to the pot experiment, Cd-spiked soil samples were subjected to three cycles of wetting (70% of water-holding capacity) and air drying to simulate the outdoor environment. The total Cd concentrations of the spiked soil samples after incubation were determined with the same digesting procedures as described above. After spiking and incubation, the total Cd concentration in Wa, Eh, Tk, and Yu was 2.85, 3.08, 3.02, and 2.96 mg/kg, respectively.

The initial concentrations of available P in the four Cd-spiked soil series were analyzed. No P was added to the control (Wa, Eh, Tk, and Yu), while in the P addition treatment (Wa + P, Eh + P, Tk + P, and Yu + P), enough P was added to increase the concentration up to at least 10 mg/kg. These treatments were replicated three times. For the P addition, a preliminary experiment was conducted before this study to identify the P amount needed to raise the available P concentration to different levels. Then, solutions of potassium phosphate were added to increase the available P concentration to at least 10 mg/kg. In total, 3.0 kg of spiked soil samples were added to each pot, and two Rhizon soil moisture samplers were vertically installed to collect soil solutions. The soil solutions were sampled at the beginning of the pot experiment, and the Cd concentrations were determined with a FAAS. Water spinach (*Ipomoea aquatic* Forsk.) seeds were sterilized for 15 min using 5% sodium hypochlorite and then sown in the potted Cd-spiked soils. The pots were translocated to a phytotron (26.2 ± 1.5 °C, relative humidity = 80.7 ± 10.1%, lighting density = 120 ± 6 μmol/sec/m^2^). Soil moisture content was determined every two to three days and maintained at 60–80% of water-holding capacity by replenishing with deionized water.

Water spinach was harvested 28 days after seeds were sown and was then divided into roots, stems, and leaves. At this time, the shoot height was measured, and the chlorophyll content of the largest extended leaf was determined using a Konica Minolta SPAD-502 and recorded as SPAD (Soil Plant Analyzer Development) readings. To remove the adsorbed Cd, the roots were first soaked in 20 mM of Na_2_-EDTA for 15 min. Fresh plant tissues were rinsed with tap water and then deionized water and mixed with the same plant tissues of the same treatment as a composite sample; then, the chemical forms of Cd in the plants were analyzed. Following Lai [6], six chemical forms were extracted in the following sequence: inorganic Cd (F_E_) extracted by 80% alcohol, water-soluble Cd (F_W_) extracted by deionized water, pectate- and protein-integrated Cd (F_NaCl_) extracted by 1 M NaCl, undissolved Cd phosphate (F_HAc_) extracted by 2% CH_3_COOH, Cd oxalate (F_HCl_) extracted by 0.6 M HCl, and residual Cd (F_R_) digested with aqua regia. All the other parts of the plants were oven dried at 65 °C for 72 h and then weighed (dry weight) before grinding with a grinder (Rong Tsong Precision Tech. Co., Taichung, Taiwan), digestion with HNO_3_/HClO_4_ (*v*/*v* = 3/1), and filtering with filter papers (Whatman No. 42). The Cd concentration in the digestant was determined using a FAAS. Soil samples were also collected at the end of the pot experiment, and the pH (*w*/*v* = 1/1) [20], electrical conductivity (EC; saturated soil pastes) [21], and available P concentration (Bray-1 method) [22] were determined after air drying, grinding, and passing through 10-mesh stainless steel sieves.

Because the sequence extraction was conducted for the analysis of chemical form in the composite sample, recovery rates were calculated based on the ratios between the sum of six chemical forms and the total Cd concentration. The data were considered valid when the recovery rate was 90–110%. Statistical analysis was performed using the Statistical Package for the Social Sciences (SPSS, Armonk, NY, USA). Analysis of variance (ANOVA) was used to test the effect of P treatment on the Cd concentration of the soil solution, pH, EC, available P concentration, shoot height, SPAD reading, and dry weight. Treatments were compared with the least significant difference (LSD) test and paired *t*-test at with significance level of *p* < 0.05.

## 3. Results and Discussion

### 3.1. Soil Properties and Cd in Soil Solutions

**Figure 1:**
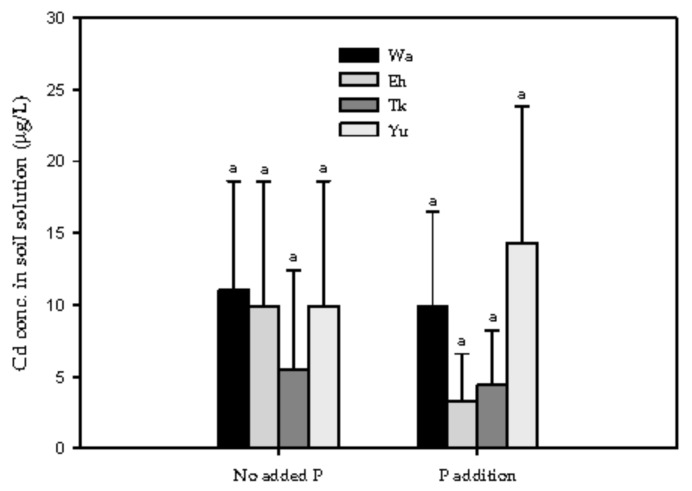
Effect of adding P on the Cd concentrations of the soil solutions. The same lowercase letter indicates no significant differences between the soil series for the same treatment. Replicates (*n*) = 3.

The Cd concentrations of the soil solutions collected before the pot experiment were all less than 0.015 mg/L (Figure 1). By multiplying the solutions’ Cd concentration, water-holding capacity, and soil weight per pot, the total Cd mass in the soil solution was found to be less than 0.015 mg per pot (<0.2% of the total Cd), indicating that the availability of spiked Cd was quite low.

Except for the available P concentration, there was no significant influence of P addition on the pH or EC of the four soil series (Table 2). Relative to Wa and Tk, Eh and Yu had higher EC values, and the EC of Yu + P was beyond the threshold of a saline soil (4 dS/m). The addition of P fertilizer increased the available P concentration in the four soil series beyond 10 mg/kg. For all four series, the available P concentration increased by 1–3 mg/kg over the concentration in the control after application of the fertilizer. Specifically, the addition of P significantly increased the available P concentration from 7.60 ± 0.97 (Wa) to 10.63 ± 1.31 mg/kg (Wa + P). This promoted the growth of the water spinach, but the changes in SPAD reading, shoot height, and dry weight of whole plant were not statistically significant (Table 2).

### 3.2. Cd Accumulation

**Figure 2:**
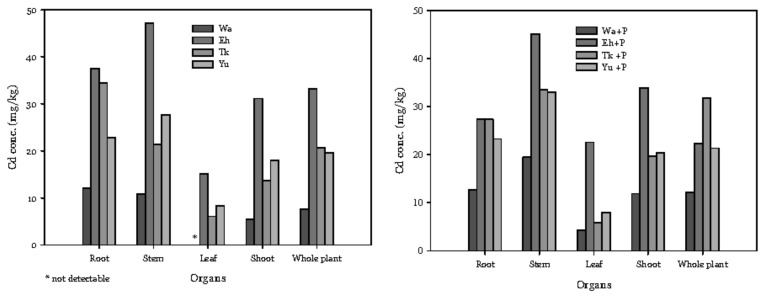
Effect of adding P on the Cd concentrations in different organs of water spinach. The meanings of the abbreviations are the same as in Table 2.

**Figure 3:**
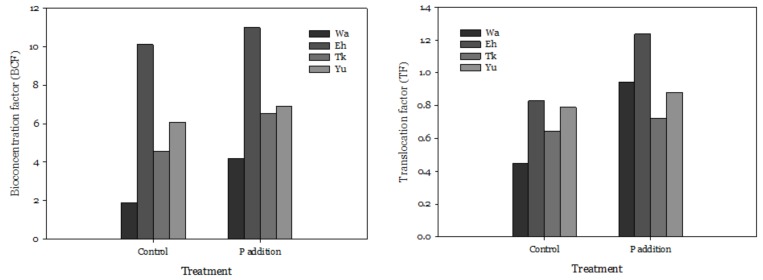
Effect of adding P on the bioconcentration factor (BCF) and translocation factor (TF) of water spinach. The meanings of the abbreviations are the same as in Table 2.

Compared with the other three soil series, there was a lower concentration of accumulated Cd in the roots (12–13 mg/kg) and shoots (5–12 mg/kg) of water spinach grown in Wa. The water spinach grown in Eh accumulated the highest concentration of Cd (Figure 2). Relative to the controls, P addition decreased Cd concentration in the roots of plants grown in Wa and Tk, but the concentration in the shoots increased 1.1 to 2.2 times. We used the bioconcentration factor (BCF; ratio of shoot concentration to soil total concentration) and translocation factor (TF; ratio of shoot concentration to root concentration) to assess the upward translocation of Cd. Treatment of Eh and Eh + P had higher BCF and TF in comparison with the other three soil series. Both the BCF and TF increased in the P treatments compared to the controls (Figure 3). The BCF of different treatments reached 1.9 to 11.0, indicating that the water spinach had a high level of Cd accumulation.

### 3.3. Chemical Form

**Figure 4:**
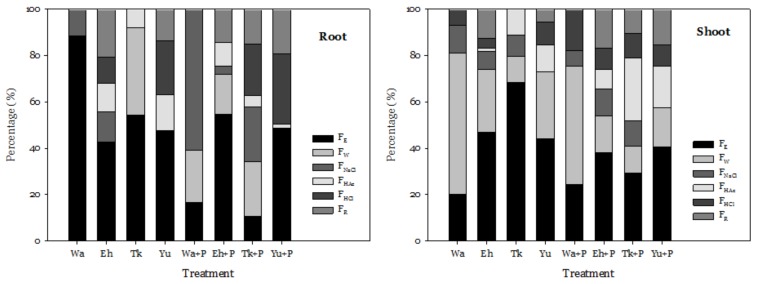
Effect of adding P on the chemical forms of Cd in the roots and shoots of water spinach. The meanings of the abbreviations are the same as in Table 2.

Most Cd accumulated in the roots of water spinach as F_E_, accounting for 43–88% of the total accumulated Cd (Figure 4). P addition did not affect the percentage of Cd in the chemical form of F_HAc_, in contrast to the results of Yin et al. [23]. However, in the Wa and Tk series, the chemical form of Cd changed from F_E_ to F_W_ and to F_NaCl_ in the P treatment. For Eh, P addition decreased the percentage of F_NaCl_, F_HAc_, F_HCl_, and F_R_.

Regardless of the treatments, the accumulated Cd was mainly compartmentalized into F_E_ and F_W_ (Figure 4). These two chemical forms have high mobility and are thus easy to translocate to other organs [4,24]. The percentage of Cd as F_E_ in shoots decreased by 6–40%, while the percentages of other chemical forms increased after the addition of P. This decreased the mobility of Cd and alleviated the toxicity of Cd [5]. P treatment also increased the percentage of F_NaCl_ in the shoots of water spinach grown in Tk and Eh. Relative to the controls, the percentage of Cd as F_HAc_ in the shoots of water spinach grown in the Tk + P, Eh + P, and Yu + P treatments increased 7–16%. In line with Yin et al. [23], for the four soil series treated with P, the sum of F_HAc_, F_HCl_, and F_R_ increased 11–37% compared to the controls. Also, similar to Du et al. [25], the experimental results revealed that P addition stabilized the accumulated Cd in the shoots, decreasing its negative effects on water spinach. This is why P addition promoted the growth of water spinach in the four soil series.

The higher percentage of Cd as F_HAc_ reveals that more CdHPO_4_, Cd_3_(PO_4_), and other compounds were formed [26]. P addition increased the percentage of Cd as F_HAc_ in shoots in Eh, and growth increased accordingly. A previous study reported that decreasing the P concentration in a solution could decrease Cd in the F_E_ or F_W_ of spinach [23]. The experimental results of this study show that the percentage of Cd as F_E_ or F_W_ was decreased in roots in Wa + P and Tk + P, and in shoots in Tk + P, Eh + P, and Yu + P. The results indicate that P addition could decrease the mobility of Cd in plants, especially shoots, grown in some soil series.

Table 3 shows the average concentrations, BCF, TF, and percentages of different chemical forms of Cd in the water spinach grown in the four soil series. Relative to the control, P addition significantly increased the Cd concentrations in the shoots. Moreover, the average of BCF and TF of the four soil series in the P treatments significantly increased 1.3 and 1.4 times in comparison with control, respectively. P addition also affected or significantly affected the proportions of different chemical forms of Cd in the roots and shoots. Even though some of the differences were not statistically significant, the average percentage of F_HAc_, F_HCl_, and F_R_ in the shoots of water spinach grown in the four soil series treated with P increased 2.1 to 2.4 times relative to the controls.

### 3.4. Health Risk Assessment

According to Antoniadis et al. [27], the vegetable-induce average daily dose (ADD_v_) and vegetable-induced hazard quotient (HQ_v_) can be calculated using Equations (1) and (2), where C_p_ is the Cd concentration (mg/kg) in the shoots of water spinach. According to the Report on the Nutrition and Health Survey in Taiwan and vegetable calorie counts, the mean individual daily vegetable consumption (MIDVC) in 2013–2016 was 0.133 kg/day. This value can be used to calculate the daily Cd intake from vegetables in relation to body weight (BW). The tolerable daily intake (TDI) used in this study is the reduced TDI—0.36 μg/kg·BW/day—set by the European Food Safety Authority [28]. Food is the dominant source of Cd exposure for humans, accounting for approximately 90% of the Cd intake [28]. Because 26% of humans’ Cd intake comes from vegetables [29], this means that the TDI from vegetables (TDI_v_) is 0.084 μg/kg·BW/day.

(1) $${\mathrm{ADD}}_{v}=\;\frac{C_{p}\;x\;\mathrm{MIDVC}}{\mathrm{kg}\cdot \mathrm{BW}}$$

(2) $${\mathrm{HQ}}_{v}=\;\frac{{\mathrm{ADD}}_{v}}{{\mathrm{TDI}}_{v}}$$

The mobility of F_E_ and F_W_ is higher than that of other chemical forms; thus, it is easy for them to translocate to other organs [4,24]. However, it is also easy for these two chemical forms to be leached out of plant tissues during the cooking process and thus not be absorbed by animals’ digestive systems. In addition to the total concentration of Cd in water spinach, the sum of the proportion of the chemical forms F_NaCl_, F_HAc_, F_HCl_, and F_R_ was used to calculate the HQ_v_, coded as HQ_v_-CF. Besides using chemical form to calculate HQ_v_, a previous study used artificial digesting agents to stimulate and assess the bioaccessibility of Cd accumulated in the edible parts of water spinach [30]. Approximately 32–55% of the Cd in water spinach is bioaccessible, meaning that it can be absorbed by the human digestive system. The median proportion of bioaccessible Cd (44%) was used to calculate the HQ_v_, coded as HQ_v_-SD.

Unlike lettuce, water spinach should be cooked before eating. In Taiwan, blanching is the most common method for cooking this green. Mnisi et al. [31] revealed that cooking removes 53.4% of the Cd in vegetables. Blanching in boiling water removes approximately 32–55% of the Cd in water spinach [30]. Based on the findings of above two studies, we assumed that 50% of the Cd in water spinach is leached into boiling water after blanching, and then used three methods to calculate the actual value of HQ_v_. Table 4 shows the ADD_v_ and HQ_v_ when the total concentration (TC), chemical form (CF), and bioaccessible fraction of Cd were considered. The HQ_v_ values were much higher than 1.0, especially in the Eh and Eh + P soil series. This result was not unexpected because the total concentration of Cd in the soil was slightly beyond the monitoring standard stipulated by the SGWPR Act of Taiwan (i.e., 2.5 mg/kg). After P treatment, the values of HQ_v_-TC, HQ_v_-CF, and HQ_v_-SD were 1.1 to 4.2 times higher compared with the controls. Previous studies have found that cooking alters the subcellular distribution and chemical form, and thus the bioaccessibility, of Cd in plant tissues [30,32,33,34]. However, this study did not assess the chemical form and proportion of bioaccessible Cd in cooked tissues under different P treatments, and thus we could not calculate the actual HQ_v_.

## 4. Conclusions

Although the soil series we examined were characterized by low availability of Cd, the grown water spinach had a high capacity to accumulate Cd. P addition changed the chemical form and mobility of Cd in the leaves, thus possibly alleviating the toxicity of Cd. The water spinach therefore accumulated and translocated more Cd to the leaves, altering the ADD_v_ and HQ_v_. The results of this study reveal that the selection of a suitable cultivar with a low capacity to accumulate Cd is needed for food security. Additional studies are needed to investigate the change in the chemical forms of Cd in other cultivars of water spinach after P treatment. Moreover, the relationship between the three methods of assessing ADD_v_ and HQ_v_ needs to be investigated further.

## Figures and Tables

**Table 1:** Selected properties of the four soil series.

Soil Series ^1^	CEC ^2^ (cmol_c_/kg)	OC ^3^ (%)	Sand (%)	Silt (%)	Clay (%)	Texture
Wa	7.23	0.84	54	17	29	Sandy clay loam
Eh	6.18	1.04	16	48	36	Silty clay loam
Tk	9.29	1.07	19	52	29	Silty clay
Yu	7.82	1.85	40	31	29	Clay loam

^1^ Wa: Wanho soil series, Eh: Erhlin soil series, TK: Taikang soil series, Yu: Yuanlin soil series; ^2^ CEC: cation exchange capacity; ^3^ OC: organic carbon content.

**Table 2:** Effect of adding P on soil properties and growth exhibition of water spinach.

Treatment ^1^	pH	EC (dS/m) ^2^	Available P Conc. (mg/kg)	SPAD Reading ^2^	Shoot Height (cm)	Dry Weight (g/plant)
Wa	7.50–7.68 a ^3,4^	1.09 ± 0.09 a	7.60 ± 0.97 b	42.9 ± 7.5 a	9.5 ± 3.9 a	0.40 ± 0.10 a
Eh	7.43–7.61 a	2.96 ± 0.22 a	10.40 ± 0.38 a	43.9 ± 6.5 a	11.1 ± 3.6 a	0.67 ± 0.21 a
Tk	7.47–7.56 a	1.29 ± 0.19 a	15.56 ± 0.42 a	40.5 ± 5.8 a	10.1 ± 4.2 a	0.47 ± 0.21 a
Yu	7.21–7.39 a	4.89 ± 1.18 a	13.34 ± 0.52 a	45.2 ± 6.1 a	8.0 ± 2.9 a	0.60 ± 0.33 a
Wa + P	7.47–7.63 a	1.32 ± 0.23 a	10.63 ± 1.31 a	39.3 ± 8.6 a	6.9 ± 2.8 a	0.48 ± 0.12 a
Eh + P	7.42–7.55 a	2.90 ± 0.31 a	12.04 ± 0.32 a	42.7 ± 5.2 a	10.0 ± 3.8 a	0.77 ± 0.38 a
Tk + P	7.43–7.54 a	1.39 ± 0.29 a	16.95 ± 2.91 a	41.5 ± 4.7 a	10.9 ± 4.3 a	0.42 ± 0.09 a
Yu + P	7.36–7.46 a	5.13 ± 0.60 a	14.43 ± 1.29 a	41.7 ± 9.2 a	9.3 ± 3.6 a	0.59 ± 0.30 a

^1^ Wa, Eh, Tk, and Yu refer to the controls, and Wa + P, Eh + P, Tk + P, and Yu + P refer to the P treatments. ^2^ EC: Electrical Conductivity; SPAD Reading: Soil Plant Analyzer Development Reading. ^3^ The same lowercase letter indicates that the effect of P treatment was not significantly different between soil series. ^4^ Mean ± standard deviation; replicates (*n*) = 3.

**Table 3:** Effect of adding P on the accumulation, translocation, and chemical form of Cd in the water spinach.

Cd in Plant	Control	P Addition
Root conc. (mg/kg)	26.73 ± 11.64 ^1^	22.67 ± 6.96
Shoot conc. (mg/kg)	17.07 ± 10.73	21.49 ± 9.11 *^,2^
Bioconcentration factor (BCF)	5.66 ± 3.43	7.15 ± 2.83 *
Translocation factor (TF)	0.68 ± 0.17	0.94 ± 0.21 *
Chemical form in the root		
F_E_ (%)	58.3 ± 20.7	32.6 ± 22.2
F_W_ (%)	9.4 ± 18.8	15.9 ± 10.9
F_NaCl_ (%)	6.1 ± 7.1	21.9 ± 28.0
F_HAc_ (%)	9.0 ± 6.8	4.3 ± 4.5
F_HCl_ (%)	8.6 ± 11.1	13.1 ± 15.5
F_R_ (%)	8.6 ± 10.4	12.2 ± 8.4
Chemical form in the shoot		
F_E_ (%)	44.8 ± 19.9	33.1 ± 7.6
F_W_ (%)	32.2 ± 21.0	23.9 ± 18.3 *
F_NaCl_ (%)	7.0 ± 5.0	7.2 ± 5.3
F_HAc_ (%)	6.1 ± 6.2	13.4 ± 11.7
F_HCl_ (%)	5.3 ± 4.3	11.8 ± 4.1 *
F_R_ (%)	4.6 ± 6.1	10.6 ± 7.6 *

^1^ Average of four soil series; Mean ± standard deviation. ^2^ The asterisk (*) indicates a significant difference between control and P addition (paired *t*-test, *p* < 0.05).

**Table 4:** Average daily dose (ADD) and hazard quotient (HQ) of the vegetable-to-human ingestion pathway (HQ_v_) in water spinach grown in contaminated soils based on different assessment methods.

Treatment ^1^	Average Daily Dose ^2^ (ADD_v_; μg/kg·BW/day)			Hazard Quotient (HQ_v_)		
	ADD_v_-TC	ADD_v_-CF	ADD_v_-SD	HQ_v_-TC	HQ_v_-CF	HQ_v_-SD
Wa	0.57	0.11	0.12	2.42	1.27	0.53
Eh	3.25	0.84	0.71	13.89	10.09	3.06
Tk	1.44	0.29	0.32	6.14	3.49	1.35
Yu	1.88	0.51	0.41	8.03	6.06	1.77
Wa + P	1.24	0.30	0.27	5.31	3.63	1.17
Eh + P	3.53	1.63	0.78	15.10	19.51	3.32
Tk + P	2.06	1.22	0.45	8.81	14.57	1.94
Yu + P	2.13	0.91	0.47	9.12	10.83	2.01

^1^ The meanings of abbreviations are the same as in Table 2. ^2^ ADD_v_-TC, ADD_v_-CF, and ADD_v_-SD were the ADD_v_ calculated based on the total concentration, chemical form, and bioaccessible fraction of Cd, respectively.
